# Interim reports for data monitoring committee review *vs* final reports for regulatory filing

**DOI:** 10.1186/1745-6215-12-S1-A36

**Published:** 2011-12-13

**Authors:** KyungMann Kim

**Affiliations:** 1 University of Wisconsin-Madison, Madison, WI 53792, USA

## 

Independent data monitoring committee is responsible for review of the ongoing safety of participants and the validity and integrity of data in clinical trials and for making recommendations to the sponsor whether the trial should continue as planned, be modified or be terminated because of safety concern, futility of the trial, or treatment benefit. Use of DMCs by government and industry sponsors has been growing steadily ever since the announcement by the US National Institutes of Health in 1998 and by the Food and Drug Administration in 2001 of policy and guidelines for data and safety monitoring.

Since a statistical analysis plan serves as a benchmark for the final analysis and the final report for regulatory filing, it needs to be explicit about its contents including the specific statistical analyses to be used. An interim analysis plan, however, serves as a benchmark for the ongoing review of the safety and efficacy data by the DMC and therefore it cannot be codified as in an SAP as it needs to be flexible to address any concerns raised by the DMC during the trial and to evolve with the emerging issues in the monitoring of the study. More importantly it needs to summarize and present the interim safety and efficacy data in the most succinct way to facilitate the review of the ongoing risk-benefit of the trial and to help the DMC members comprehend the contents of the report in the most efficient way.

Industry and contract research organization practices continue to be less than desirable in that there is a tendency to consider the interim analysis plan and resulting interim reports merely as a subset of the statistical analysis plan and the final report. This presentation will offer some thoughts on how the interim report should be prepared for DMC review and to improve the contents of the interim report.

